# Genotype–Phenotype Correlation and Therapeutic Amenability in a Cohort of Rett Syndrome Patients: A Single-Center Study

**DOI:** 10.7759/cureus.86953

**Published:** 2025-06-29

**Authors:** Rahaf Lazek, Alaa Karoum, Waseem Fathalla

**Affiliations:** 1 Pediatric Medicine, Sheikh Shakhbout Medical City, Abu dhabi, ARE; 2 Pediatric Medicine, Sheikh Shakhbout Medical City, Abu Dhabi, ARE; 3 Pediatric Neurology, Sheikh Shakhbout Medical City, Abu Dhabi, ARE

**Keywords:** gene therapy, genotype phenotype correlation, mecp2 mutation, methyl-cpg binding protein-2 (mecp2) gene, neurodevelopmental, rett syndrome, rtt, trofinetide

## Abstract

Introduction

Classical Rett syndrome (RTT) is a rare progressive neurodevelopmental disorder associated with mutations in the *MECP2* gene. This study aims to correlate the genetic mutations and phenotype characteristics of a cohort of RTT syndrome patients and evaluate their amenability to novel therapies, including Trofinetide.

Methods

We conducted a retrospective observational review of a case series (2000-2024) of RTT patients at Sheikh Shakhbout Medical City, Abu Dhabi, United Arab Emirates (ARE). We included patients under 16 years of age with classic RTT and a confirmed *MECP2* mutation. We analyzed demographic, clinical, and genetic data to determine genotype-phenotype correlations.

Results

Of 13 patients with RTT syndrome, 8 patients had genetic data on record and were included in the phenotype-genotype correlation analysis; the entire cohort (n=13) was included in the clinical profiling. The age at presentation was 11.2 years, and the median age was 23 months. The distribution of patients’ stages at presentation was as follows: 7(53%) were in stage I, 5 (38%) were in stage II, and 1 (8%) were in stage III. Progression to Stages III and IV took place collectively in 9 (75%) of patients.

Of the eight patients with genetic data, 6/8 (75%) had variants classified as pathogenic/likely pathogenic, whereas 2/8 (25%) had variants classified as variants of unknown significance (VUS) or benign, however, both met clinical diagnostic criteria for RTT and were assessed by the authors and treating team as pathogenic. These two novel variants were in two classical patients with RTT, classified as VUS (1330G>A) and benign (641C>A).

The cohort revealed a high frequency of comorbidities, including epilepsy 8 (61%), behavioral disturbances 7 (53%), gastrointestinal complications 6 (46%), and scoliosis 4 (30%). 5 (43%) of patients had MRI abnormalities. As for treatment amenability, all patients in our cohort are eligible for the only currently approved treatment (Trofinetide), regardless of genotype-phenotype correlation.

Conclusion

This study demonstrates the clinical and genetic heterogeneity in RTT, and the value of genotyping for confirmation, assessment, and management planning. While no genotype-phenotype profile excludes treatment eligibility with Trofinetide, this correlation may inform early life response to treatment and eligibility for other emerging therapeutic options such as gene therapy. Early recognition, diagnosis, and treatment will certainly have an impact on patient outcomes.

## Introduction

This study was previously presented as a poster at the 19th SEHA International Pediatric Conference on 21-23 February, 2025.

Rett syndrome (RTT; OMIM 312750) is a rare, progressive, neurodevelopmental, X-linked dominant disorder [1, 2]. Almost all cases are caused by de novo mutations [1], with a global prevalence of 1 in 10,000 to 15,000 females [2].

Typical RTT is associated with mutations in the gene coding for the methyl-CpG-binding protein 2 (MECP2), located on the Xq28 region of the X chromosome. MECP2 is a DNA-binding protein that functions as both a transcriptional regulator and repressor [1]. Deficiency of MECP2 can lead to disruptions in both neuronal maturation and synaptic plasticity [3-5]. Most MECP2 mutations are single-nucleotide substitutions or small insertions and deletions. Recent studies have shown that the *MECP2 *gene is prone to significant rearrangements, such as duplications and deletions of complete exons, primarily affecting exons 3 and 4 or perhaps the entire gene [6, 7].

The clinical diagnosis of RTT depends on a set of clearly defined inclusive and exclusive criteria [8-10]. Individuals with the syndrome undergo a period of normal neurological and physical development during the first 6-18 months of life [8, 11]. The first features of RTT begin with failure to reach developmental milestones, manifesting in early childhood and progressing through four stages: Stage I: stagnation (age 6-18 months), stage II: rapid regression (age 1-4 years) characterized by deceleration of head growth, loss of acquired purposeful hand skills and spoken language, repetitive stereotypic hand movement, and delays in motor milestones, stage III: pseudostationary (age 2 years to adulthood) with no continued skill regression, characterized by stabilization of symptoms and slight improvement in some skills, and finally stage IV: late motor deterioration stage (typically after age 10), progressive decline in motor function, loss of mobility, scoliosis, and muscle weakness [8]. Other organ system involvement provides supportive criteria such as disorganized breathing patterns, autonomic and cardiac abnormalities, sleep disturbances, scoliosis, seizures, and gastrointestinal abnormalities, disinterest in social interaction, and unique intense eye communication “eye pointing” [10, 12-14].

Trofinetide, the first drug approved by the U.S. Food and Drug Administration (FDA) for the treatment of RTT, is an analog of insulin-like growth factor-1 (IGF1). It enhances the expression of genes associated with synaptic function and improves synaptic plasticity in RTT animal models [15-17], with the aim of restoring brain cell function and hence improving the burden of core symptoms.

The present study explores the genetic mutations and related phenotypic features of a cohort of 13 females with RTT and evaluates their potential eligibility for the novel treatment with Trofinetide.

## Materials and methods

A retrospective observational study was conducted from 2000 to 2024. Data was collected from patients’ electronic medical records. We used the International Statistical Classification of Diseases and Related Health Problems (ICD), version 9 (ICD-9 code 330.8), and ICD version 10 (ICD-10 code F84.2), to extract the data of patients with RTT who presented to the pediatric neurology service within the last 10 years at Sheikh Shakhbout Medical City, Abu Dhabi, ARE. The inclusion criteria were a confirmed diagnosis of RTT under 16 years of age at first presentation with available genetic data. Exclusion criteria included male sex, Rett-like syndrome caused by other mutations, and absence of genetic data.

A total of 17 patients were identified. Four were excluded: two due to atypical Rett-like syndrome with FOXG1 or CDKL5 mutations, one due to male sex, and one due to the absence of genetic data.

**Figure 1:**
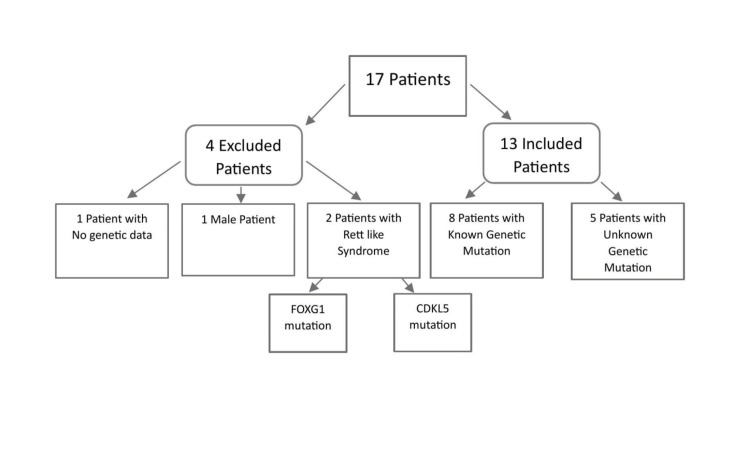
Flowchart showing the summary of inclusion and exclusion criteria for the research cohort. Of the 17 cases investigated, 4 were eliminated for the following reasons: missing genetic data (1 patient), male gender (1 patient), and Rett-like syndrome due to FOXP1 or CDKL5 mutations (2 patients). 13 RTT patients were included in the study, representing 8 with confirmed MECP2 mutations and 5 with unknown genetic mutations.

For the 13 patients who met our inclusion criteria, we examined their demographic data, clinical presentation, staging, genotypes, age at onset, and age at diagnosis (Figure 1).

## Results

We analyzed 13 patients currently aged 3 to 18 years (mean age: 11.2 years) who were diagnosed with the classical form of RTT. All our patients were UAE residents; 12 (92%) were UAE nationals, and one (8%) was an expatriate. All 13 patients fulfilled the diagnostic criteria for classical RTT. The age at first presentation ranged from 11 months to 4 years, with a median age of 23 months.

Upon initial presentation, seven (53%) of patients were in stage one, five (38%) were in stage 2, while only one (8%) presented late in stage 3. The time from first presentation to last visit ranged from 13 months to 17 years, with an average of 8 years and 5 months. On their last visit to our facility, most had progressed to stages 3 and 4 of the disease, 6 (46%) and 4 (30%), respectively. Two (15%) patients lost follow-up, and one patient (7%) remained in stage one.

RTT was suspected in 8 of the 13 cases, 62.5% at the time of presentation, given their classical features, for which *MECP2 *single-gene testing sequencing analysis was ordered.

Genetic testing details were available for 8 of the 13 patients; the remaining five had documentation of genetic confirmation, but no genotype data was accessible (Table 1). Single-nucleotide variants (SNVs) mutation in the *MECP2 *gene were identified in 6 (75%), while 2 (25%) had either an early termination codon or a frameshift mutation leading to a truncated protein. Of the six patients with point mutations, 4 (66.7%) were classified by the reference lab as pathogenic, while 2 (33.3%) were classified as benign or a variant with unknown significance (VUS).

**Table 1:** The patient’s genotype and its clinical interpretation. WES: whole exome sequencing; MECP2: methyl-CpG-binding protein 2; MBD: methyl-CpG binding domain; TRD: transcriptional repression domain; NLS: nuclear localization signal; C-terminal: carboxy-terminal; del: deletion; >: substitution

Patient ID	Study type	Nucleotide change	Protein change	Domain	Effect	Clinical interpretation
A	WES	397C>T	R133C	MBD	Missense mutation	Pathogenic
B	MECP2 sequencing analysis	473C>T	T158M	MBD	Missense mutation	Pathogenic
C	MECP2 sequencing analysis	916C>T	R306C	TRD	Missense mutation	Pathogenic
D	MECP2sequencing analysis	502C>T	R168X	NLS	Nonsense Mutation	Pathogenic
E	WES	763C>T	R255X	TRD	Nonsense mutation	Pathogenic/Likely pathogenic
F	WES	1200_1243del	P401X	c-terminal	Frameshift	Pathogenic
G	MECP2 sequencing analysis	1330G>A	A444T	c-terminal	Unknown	Unknown significance/Benign
H	MECP2 sequencing analysis	641C>A	A214	Unknown	Unknown	Benign

Comorbidities with RTT

At the first presentation, three (30%) of our patients had microcephaly, and five (38%) had dysmorphic features. Moreover, eight (61%) of patients developed seizures. Among those with seizures, 7 (87.5%) had intractable epilepsy. Interestingly, one patient had seizures at the first presentation and was found to have agenesis of the corpus callosum along with other comorbidities. Electroencephalograms (EEGs) were abnormal in all patients with seizures.

Behavioral disturbances were observed in 7 (53%), and included autistic behavior, attention-deficit/hyperactivity disorder (ADHD), or self-mutilation, staring spells, and impulsivity. Sleep disturbances were observed in 4 (30%), with most of them treated with melatonin. One patient needed clonidine as an adjunctive medication. Gastrointestinal comorbidities were present in approximately one-third to half of the patients: 4 (30%) had feeding problems and dysphagia, while 6 (46%) had constipation. Only 2 (15%) of patients were screened for cardiac abnormalities. Abnormal breathing patterns such as breath-holding or hyperventilation episodes were found in 4 (30%) of patients. Similarly, 4 (30%) had scoliosis. Four of our patients demonstrated normal brain MRI, whereas two had wide perivascular spaces, and one had agenesis of the corpus callosum.

## Discussion

Phenotypic presentation

The most common presentation in our patient cohort was delayed developmental milestones. Our cohort had a high rate of comorbidities at presentation; most of our patients exhibited feeding problems and dysphagia, with a higher rate of constipation. These findings are consistent with previous studies, which reported a greater proportion of gastrointestinal and feeding dependence, particularly in younger patients, with a decrease in symptom prevalence observed in older individuals [18, 19].

While reviewing the findings of epilepsy studies among RTT, it becomes evident that seizures are a prevalent and challenging aspect of the condition. Our study revealed a substantial proportion of patients experiencing seizures. In contrast to the study by Zade et al., showing that a minority of their patients reported to be resistant to anti-seizure medications [20]. While others reported that seizures were found in 63% of patients before age 5, and in 21.4% of cases, treatment did not effectively control seizures [21]. It is well known that certain mutations are associated with lower rates of epileptic seizures, such as the p.R255X mutation [22-24]. One patient with p.R255X mutations was identified in our cohort, a 3-year-old girl who did not experience seizures. In our patient cohort, however, crucial information regarding the nature of epilepsy, seizure types, and age of onset was lacking.

Behavioral manifestations are prevalent among individuals with RTT syndrome, as evidenced by findings from both the Ireland study [20] and our own. In the Ireland study, excitement was commonly reported, especially in younger individuals, whereas autistic behavior was predominant in our patient cohort. Notably, sadness was not observed in our study, contrasting with the Ireland findings. However, both studies noted the occurrence of self-injury tendencies among participants [20]. Almost all individuals with RTT exhibit mild to moderate internalizing tendencies. Participants with mixed (internalizing and externalizing) behaviors were younger in age, with a mild MECP2 mutation and good motor function; they tend to have more prominent behavioral problems with worsening behaviors over time [25].

Scoliosis is a major skeletal abnormality in RTT; in a cohort of patients with RTT syndrome (n=554), scoliosis was reported (n=292) in almost 50%, 13% of whom underwent scoliosis surgery [26]. In another study, around 30% had severe scoliosis, 18% of whom had scoliosis surgery [27]. None of the patients in our study required corrective scoliosis surgery in our institution; however, two out of four patients (50%) had surgery elsewhere (records not available), which is a higher percentage compared to surgical correction performed in other studies [26, 27]. The MECP2 mutations R255X and R168X have been associated with a higher incidence of scoliosis, while R133X is associated with a lower incidence [28]. Of interest, our patient with the R255X mutation developed scoliosis at the age of 3 years, consistent with a severe phenotype.

Breathing abnormalities ranging from breath-holding to hyperventilation were found in 64% of individuals in one study [20], while such abnormalities were less frequently reported in our cohort. Recognizing such events is important since they are commonly mistaken for seizures. EEG correlates of these non-epileptic events may show diffuse, high-amplitude slowing or changes associated with hyperventilation, which might be misinterpreted as ictal electrographic alterations. The severity of episodes typically determines the extent of EEG activity. While the exact mechanism of abnormal breathing and its association with the MECP2 mutation remains unclear, it may involve impaired neuromodulation and synaptic transmission in the brainstem respiratory regions [29, 30].

Microcephaly is a cardinal feature of RTT. During the first year of life, head development slows down, followed by somatic growth (height/weight) declining. In a postmortem study, reduced occipitofrontal circumference (OFC) reflected smaller brain size, and cortical neuron dendrite measurements indicated cessation in development and growth [31]. Radiological studies also showed structural changes in the brain [32]; however, the finding of agenesis of corpus callosum and wide perivascular spaces in some of our patients is not typically reported in RTT syndrome. In our patient cohort, weight loss was not observed, while a study conducted in Brazil revealed weight deficits in 37.0% of participants, with 11.1% of them classified as overweight [18].

Genotype

Our study highlights the importance of accurate phenotyping for the proper interpretation of *MECP2 *gene variants classified as VUS or even benign. A secondary aim, attempted in the present study, is to explore genotype-phenotype correlation among patients who have an MECP2 mutation.

In our study, seven (87%) of patients had an SNV in the *MECP2 *gene, while only one patient (13%) had a deletion (Table 1). A total of eight different mutations were detected, two of which, c.641C>A and c.1330G>A, have not been previously described as pathogenic mutations. Regarding mutations affecting the protein sequence, 2 (25%) of the RTT-causing SNVs were truncating, changing Arginine into a stop codon, in comparison to a study reporting half of their mutations as such, Arginine is changed into a Cysteine in another 2 (25%) of our patients, this substitution causes major changes in protein 3D structure [33].

Our results agree that most mutations are C>T transitions located in CpG hotspots [34-40]. The CpG dinucleotide is the site of DNA methylation, and its methylation contributes to gene silencing and changes in chromatin structure. Five (62%) of the mutations observed in our cohort (patients 1, 3, 4, 6, and 8) have been frequently reported before in RTT studies and are considered among the most common pathogenic variants [33].

Genotype-phenotype correlation

It has been hypothesized that distinct mutation types are associated with varying degrees of clinical severity. Three clinical variables appear to be key contributors to this heterogeneity: ambulation, hand usage, and language. Individuals with the R168X mutation have reduced hand function compared to R133C mutation, while the latter tend to preserve better language abilities [41]. For instance, a study revealed that a patient with the mutation R168X began regressing when she was five months old and suffered progressive deterioration [42]. Our patient with the R168X mutation first showed signs of regression when at 20 months old; currently, she is at stage IV of the disease, indicating a severe phenotype. This finding is consistent with prior studies [21]. Again, early presentation may correlate with the severity of the disease (refer to the progression chart).

Furthermore, mutations that disrupt the nuclear localization signal (NLS) domain of the *MECP2 *gene or result in early truncation are linked to a more severe phenotype than missense mutations, while C-terminal deletions tend to result in milder features [43]. Girls with the R255X mutation tend to have severe disease [44], which is evident in our patient; she is nonverbal, has a wide-based gait, and lacks hand dexterity. On the contrary, the R133 mutation is mostly associated with better overall function, less hand stereotypy, and preserved ambulation [21, 45-47]. Our findings: our 12-year-old patient (E), who has stage III RTT, has a wide base gait, is non-verbal, has epilepsy, and behavioral problems. The fact that pathogenic missense mutations in the methyl-CpG-binding (MBD) domain (see Table 1) result in decreased binding to methylated DNA emphasized the functional significance of this area [48].

Females with the R306C mutation tend to retain their ability to walk [49], similar to what we had observed. However, other studies have reported that this mutation is associated with an intermediate severity phenotype [21].

Patient H, who carries the A214 benign mutation, first presented at age five at stage II. Initially, she exhibited developmental delay with autistic features and ADHD, was nonverbal, but had a normal gait. Gradually, she began to display repetitive hand movements, self-mutilation behavior, constipation, poor eye contact, limited social interaction, and sleep disturbances. At eight years, she was diagnosed with RTT syndrome. At age ten, she displayed aggressive behavior and was prescribed risperidone. EEG results showed no epileptic discharge. At age 16, she was at RTT stage III, was thriving well without developing seizures or abnormal respiratory patterns, and retained the ability to eat orally and walk with a steady gait.

**Figure 2:**
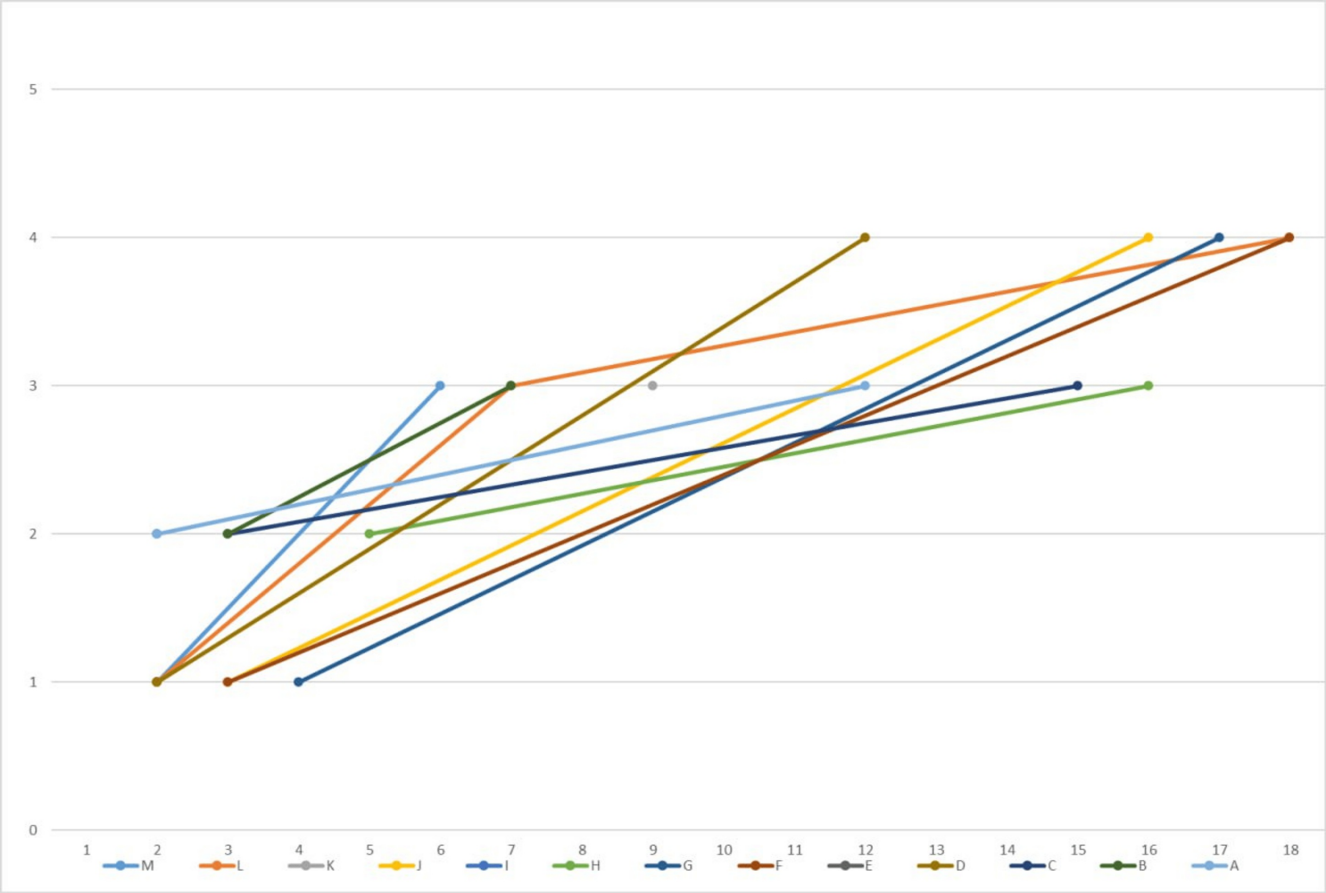
This line graph illustrates the RTT patients’ stage at the time of presentation and at the last visit. The X-axis represents the age of the patients in years, while the Y-axis represents the stage. Each colored line shows individual patients. The lines were plotted against their progression over time. Most of our cases were at stage 3 or 4 of disease advancement at the last visit. We have three of our cases, E, I, and K only visited us once.

The A444T polymorphism was classified as a benign variant (Buyse et al.) based on parental analysis demonstrating the variant in the unaffected father [50]. Coutinho et al. also reported - in a study done on 172 unrelated autistic children - the same variant as polymorphism [51]. A study performed in the Czech Republic identified four cases with this mutation, one with classic RTT and three having Rett-like features [48]. However, the Rett database does not report this polymorphism as pathogenic [6]. On the contrary, our patient, who presented at 4 years old, manifested global developmental delay, corpus callosum agenesis, epilepsy, and midline hand wringing movements. She is currently at stage IV, in a wheelchair, hypotonic, and displays self-mutilation, autistic features, insomnia, and irritability (Figure 2). Generally, benign variants are most likely to be silent, with no amino acid changes or altered protein structure. There may be several factors that may explain why this gene variant led to RTT phenotype in some individuals while being reported as benign in others. One of the explanations is that the RTT phenotype expression is modulated by X-inactivation [52]. Another explanation could be the presence of modifier gene effects [53], potentially explaining the severity and variability of the phenotype, and leading to genotype-phenotype correlation inconsistency. Females who have a MECP2 pathogenic variant with favorably skewed XCI exhibit mild or no manifestations of the disease. However, some studies have stated that XCI mosaicism in the RTT population is not significantly different from the normal population, with approximately 85-90% of all individuals exhibiting a random pattern of X-inactivation [40].

Amenability for treatment

Approved Therapies

Trofinetide was FDA approved based on the LAVENDER study (NCT04181723) for patients with genetically confirmed RTT syndrome who are above 2 years of age. In our cohort, all our patients are candidates for treatment with Trofinetide; we would have to establish our patients’ clinical scale rating (Clinical Global Impression-Severity (CGI-S) and Rett Syndrome Behavioral Questionnaire (RSBQ) can be used to assess their responsiveness to this treatment. Alternatively, real-life experience post marketing may provide further insight into response patterns among different patients. It is logically predicted that earlier treatment will have a better outcome.

Pipeline Therapies

The NGN-401 gene therapy, a recombinant AAV9 containing a full-length human *MECP2 *gene, is actively recruiting females aged 4 to 10 years with typical RTT [54]. The treatment aims to express therapeutic levels of the MECP2 protein without causing overexpression. Among our cohort, four out of 13 patients met the inclusion criteria. The remaining patients would be excluded due to comorbid genetic conditions in addition to the deletion in MECP2. An additional patient would be excluded because she has agenesis of the corpus callosum, as already discussed. The rest of the seven patients would be excluded due to age.

In another gene therapy clinical trial using CRISPR/Cas9-based technology, four patients from our cohort would meet the inclusion criteria: those who carried T158M, R168X, R255X, and R306C [55].

A multicenter study of TSHA-102, an investigational gene therapy, is also actively recruiting typical RTT patients from 5-8 years of age; only three of our cohort would meet inclusion criteria [56].

This study has multiple limitations. Initially, it is a retrospective, single-center study with a small sample size; the generalizability of the results may be limited. Additionally, incomplete genotype data for five patients reduced the strength of genotype-phenotype correlation analysis. Some clinical data, such as detailed seizure characterization, were incomplete due to inappropriate medical record documentation. Future prospective studies with a larger sample size are important to address those limitations.

## Conclusions

Pathogenic mutations in MECP2 exhibit significant overlap. Although their predictive relevance for individuals with RTT is limited, these data can provide a broad indicator of phenotypic severity in counseling families. The differences in phenotype severity can result from genetic variables, such as X-inactivation skewing in the CNS, or due to medical interventions such as speech, language, and physiotherapy.

The therapeutic landscape of classic MECP2-related RTT syndrome is changing, with now approved treatment already available.

Early recognition of genetic confirmation is crucial for optimal outcome and response to therapy. The wide phenotypic severity warrants good assessment & genotype-phenotype prediction to provide the best counseling and predict response to therapy or potentially choose the best therapy once other treatments are approved.

Our study provides real-life experience that can guide assessment and decision-making during this exciting era of RTT management.
